# Long-Term CD4^+^ T-Cell and Immunoglobulin G Immune Responses in Oncology Workers following COVID-19 Vaccination: An Interim Analysis of a Prospective Cohort Study

**DOI:** 10.3390/vaccines10111931

**Published:** 2022-11-15

**Authors:** Corey Gallen, Christopher W. Dukes, Amy Aldrich, Lauren Macaisa, Qianxing Mo, Christopher L. Cubitt, Shari Pilon-Thomas, Anna R. Giuliano, Brian J. Czerniecki, Ricardo L. B. Costa

**Affiliations:** 1 Department of Clinical Science, Moffitt Cancer Center, Tampa, FL 33612, USA; 2 Department of Immunology, Moffitt Cancer Center, Tampa, FL 33612, USA; 3 Department of Biostatistics and Bioinformatics, Moffitt Cancer Center, Tampa, FL 33612, USA; 4 Immune Monitoring Core Facility, Moffitt Cancer Center, Tampa, FL 33612, USA; 5 Center for Immunization and Infection Research in Cancer, Moffitt Cancer Center, Tampa, FL 33612, USA; 6 Department of Cancer Epidemiology, Moffitt Cancer Center, Tampa, FL 33612, USA; 7 Department of Breast Oncology, Moffitt Cancer Center, Tampa, FL 33612, USA

**Keywords:** SARS-CoV-2, COVID-19, vaccine, immunity, delta, omicron, oncology workers

## Abstract

We conducted a prospective study to evaluate immune responses to SARS-CoV-2 in oncology workers in which we collected blood and clinical data every 6 months. Spike-specific CD4^+^ T-cells and immunoglobulin G responses were measured using interferon-gamma enzyme-linked immunosorbent spot and enzyme-linked immunosorbent assay, respectively. Sixty (81%) vaccinated and 14 (19%) unvaccinated individuals were enrolled. CD4^+^ T-cell responses of those individuals currently naturally infected were comparable to those who were 6 months from receiving their last dose of the vaccine; both responses were significantly higher than among those who were unvaccinated. Unvaccinated participants who became vaccinated while in the study showed a significant increase in both types of spike-specific immune responses. Previously vaccinated individuals who received a third dose (booster) showed a similar response to the spike protein. However, this response decreases as soon as 3 months but does not dip below the established response following two doses. Response to variants of concern B.1.617.2 (Delta) and B.1.1.529 (Omicron) also increased, with the Omicron variant having a significantly lower response when compared to Delta and the wild type. We conclude that antibody and T-cell responses increase in oncology workers after serial vaccination but can wane over time

## 1. Introduction

Since December 2019, the world has been challenged with an unprecedented pandemic by the number of humans affected, the velocity of spread, and the global distribution of cases. COVID-19 disease is caused by a single-strand RNA coronavirus: SARS-CoV-2. In only 12 months, more than 70 million persons were infected and 1.6 million died from COVID-19 [1]. In addition to these staggering measures of morbidity and mortality, societal and economic burdens are severe. Developmental therapeutics reacted accordingly; initially, RNA-based vaccines coding the spike protein (S protein) of SARS-CoV-2 have been shown to elicit humoral and cellular immune responses [2,3,4]. It has been previously shown that these vaccines not only formulate a robust antibody response to the S protein but also prime T-cells [5,6]. More specifically, spike-specific CD4^+^ T-cells increase after just one dose, while spike-specific CD8^+^ T-cells develop more gradually and are much more variable [5]. Robust T-cell responses in other mRNA vaccines have been shown to be associated with long-term protection against other viral infections and served as surrogate endpoints in early clinical trials aimed to develop currently approved vaccines against severe COVID-19 [7]. Subsequently, between 27 July 2020, and 14 November 2020, a total of 43,448 healthy participants were treated in a prospective international trial assessing the efficacy and safety of an mRNA-based COVID-19 vaccine (BNT162b2; a lipid nanoparticle-formulated, nucleoside-modified RNA); this vaccine was 95% effective in preventing severe COVID-19 among participants (95% CI, 90.3–97.6) [8]. Since then, based on high short-term efficacy, a total of four vaccines have been approved by the FDA for preventing COVID-19; BNT162b2 (Pfizer-BioNTech, mRNA, New York, NY, USA), mRNA-1273 (Moderna, mRNA, Cambridge, MA, USA), NVX-CoV2373 (Novavax, adjuvanted recombinant protein, Gaithersburg, ML, USA), and Ad26.COV2.S (Johnson & Johnson–Janssen, viral vector, Titusville, NY, USA), [8,9,10,11]. Notwithstanding the benefit of vaccines, real-world data already support that immune protection wanes over time likely as a function of both declines in immune activation and the emergence of new SARS-CoV-2 variants (e.g., Delta and Omicron) [12]. As a corollary, there is a clear knowledge gap about the longevity of immune activation elicited by approved vaccines in high-risk populations. This is especially important to oncology workers who are at a particularly high absolute risk of COVID-19 infection due to increased contact with a high number of patients with compromised immune systems. In this prospective cohort study, we followed vaccinated oncology workers to assess the risk of COVID-19 alongside longitudinal measures of immune activation. Herein, we present the preliminary results after 12 months after the study entry.

## 2. Materials and Methods

### 2.1. Data and Blood-Sample Collection

Participants were informed of the opportunity to participate in this study at Moffitt Cancer Center (MCC) in Tampa, Florida, United States by posting the study opening and design on the MCC external webpage, available to the general public. Inclusion criteria included vaccinated workers (completed a vaccination regimen according to FDA-approved label) in oncology health care, aged > 18, who were willing to visit MCC for study participation. We also enrolled participants who initially declined the recommended vaccination and were never vaccinated against SARS-CoV-2 at study entry. Clinical data and blood samples were collected at five time points (t1 [up to 6 months from the last COVID-19 vaccine injection] and t2–5 [every 6 months ± 1 month]) for 24 months. This report is an interim analysis of the first two time points along with added collections after additional vaccinations, such as booster doses and initial vaccination after study entry (these collections were performed within 2 weeks after study entry). After individuals consented to participate, we verified the dates of their COVID-19 vaccinations by checking their vaccination cards; side effects or complications were documented by reviewing their medical histories. Serious complications or side effects from COVID-19 or vaccination, respectively, were defined as a clinical scenario leading to hospitalization, prolongation of hospitalization, and the development of persistent or significant disability. We documented participants’ age, race, gender, history of COVID-19, chronic use of oral steroids, chronic use of immunosuppressants, and diagnosis of autoimmune disease. We also documented participants’ risk of developing COVID-19 based on exposure to patients as patient-facing vs nonpatient-facing job posts within an oncology setting. At baseline and each follow-up visit, we collected blood from participants and presented them with a questionnaire to collect dates of subsequent boosters, new diagnoses after study entry, and occurrence of COVID-19 disease after study entry. A confirmed COVID-19 infection was defined according to the FDA criteria as the presence of at least one of the following symptoms: fever, new or increased cough, new or increased shortness of breath, chills, new or increased muscle pain, new loss of taste or smell, sore throat, diarrhea, or vomiting, combined with a respiratory specimen obtained during the symptomatic period or within 4 days before or after it that was positive for SARS-CoV-2 by nucleic acid amplification-based testing and/or antigen testing. Epidemiological and clinical data are collated into a single database (REDCap), and laboratory data are collated into a single database, all after de-identification. All data are stored on password-protected computers. Access to the files is limited to the investigators and collaborators listed in the study protocol. The study conduct and all language material on the study webpage posting and clinical questionnaires were IRB-approved before the study initiation. This study was conducted in harmony with the MCC guideline for conducting nontherapeutic research, which kept both study participants and research staff at minimal risk of COVID-19. Finally, we used historical data from a different cohort of 45 unvaccinated individuals with a documented confirmed diagnosis of COVID-19 and blood collected between October 2020 and March 2021 shortly after each participant’s diagnosis for baseline comparisons of immune activation as detailed by Giuliano et al. [13]. A schematic representation of cohorts and the timing of blood collection is shown in Supplementary Figure S1.

### 2.2. Isolation of Peripheral Blood Mononuclear Cells and Serum from Whole Blood

Peripheral blood mononuclear cells (PBMCs) were isolated using BD Vacutainer CPT-cell Preparation Tubes with sodium heparin following the manufacturer’s instructions. The serum was isolated using BD Vacutainer SST Tubes following the manufacturer’s instructions.

### 2.3. Interferon Gamma Enzyme-Linked Immunospot Assay

Interferon gamma (IFN-γ) secreted from CD4^+^ T-cells was measured via enzyme-linked immunospot assays (ELISpot) of PBMCs pulsed with the full-length wild-type SARS-CoV-2 S protein. Secreted IFN-γ was captured on precoated membrane immunospot plates (Cellular Technologies Limited, Human IFN-γ ELISpot Kit, Shaker Heights, OH, USA). Cryopreserved PBMCs were thawed and washed before being plated (2 × 10^5^ cells/mL) in triplicate using CTL-Test media (provided with the kit) supplemented with 1% L-glutamine and cultured with either S protein (500 mg/mL), an anti-CD3 positive control (Orthoclone OKT3, Johnson and Johnson, cat. No. 73337989) or left unstimulated. The cells were then incubated for 48 h (37 °C, 5% CO_2_) with low humidity and processed according to the manufacturer’s instructions. In brief, plates were washed, and the detection antibody (anti-human IFN-γ Biotin; 100 mg/mL) was added to each well. After incubation at room temperature for 2 h, 1:1000-diluted streptavidin-AP was added and incubated for 30 min. Plates were washed again before substrate solution was added, and plates were incubated for 15 min to allow for color development and washed with tap water and dried overnight at room temperature. Spot-forming cells (SFCs) were counted and quality controlled (i.e., removed false positives) using CTL Immunospot Analyzer (Cellular Technologies Limited, Shaker Heights, OH, USA). The mean of each triplicate was calculated. To correct for background, the mean of SFC in the unstimulated wells was subtracted from the mean of each triplicate. The same methods were followed when measuring response to full-length recombinant S-protein variants B.1.617.2 (Delta, R&D Systems, cat. No. 10942-CV-100, Minneapolis, MN, USA) and B.1.1.529 (Omicron, R&D Biosystems, cat. No. 11060-CV-100).

### 2.4. Immunoglobulin G Antibody Enzyme-Linked Immunosorbent Assay

Serostatus of the samples was evaluated using the 384-well enzyme-linked immunosorbent assay (ELISA) adapted from the Krammer protocol as described by Giuliano et al. [14,15,16]. Positive and negative controls included convalescent serum from persons confirmed SARS-CoV-2 seropositive by ELISA and serum pools from persons confirmed SARS-CoV-2 seronegative by ELISA and were collected before 2020, respectively. Antibody concentrations were quantified using the Human SARS-CoV-2 Serology Standard provided by the Frederick National Laboratory for Cancer Research.

### 2.5. Antibody Response to SARS-CoV-2 Variants

Serostatus was evaluated using a two-step ELISA adapted from the Krammer protocol and described by Giuliano et al. [13,14,15,16]. Samples were tested against the full-length wild-type S protein in 10 threefold dilutions. To compare against emerging S-protein variants, samples were also tested for serostatus against full-length Delta variant and Omicron variant S proteins. Samples that had two consecutive dilutions with optical density values greater than triple the standard deviation of the mean of the negative controls were determined to be positive. Negative controls included serum from persons collected before 2020 and confirmed SARS-CoV-2 seronegative on ELISA. Positive controls included convalescent serum from persons with confirmed SARS-CoV-2 infection.

### 2.6. HLA Antigen Class I– and II–Blocking

Cryopreserved PBMCs were thawed before being resuspended in media provided (i.e., Cellular Technologies Limited, Shaker Heights, OH, USA) with Human IFN-γ ELISpot Kit supplemented with 1% L-glutamine. Either anti-HLAI (anti-HLA-A,B,C, clone W6/32, Biolegend cat. 311402), anti-HLAII (anti-HLA-DR,DP,DQ, clone Tü39, Biolegend cat. 361702) or an isotype control (mouse immunoglobulin G [IgG] 2a, κ, clone MOPC-173, Biolegend, cat. 400202) were added at 20µg/mL and incubated at 37 °C, 5% CO_2_ with low humidity for 1 h. Afterward, cells were used in an ELISpot assay as described previously.

### 2.7. Endpoints and Statistical Methods

The sample size for this observational prospective study was not driven by a hypothesis but rather by participant availability. The primary endpoint for this interim analysis is to assess serum IgG titers and CD4^+^ T-cell (ELISpot) immune response against COVID-19 S protein at two time points in 6 months, with additional time points following additional vaccination doses. The secondary endpoints included assessing the absolute risk of COVID-19 infection among vaccinated and unvaccinated participants and the exploration of associations between immune responses and COVID-19 infection, alongside the associations between immune responses and variables of interest pertaining to participant-related characteristics. The categorical outcomes were summarized by counts and proportions of subjects with 95% exact CI in corresponding groups. Analyses were made in GraphPad Prism version 9.0 (GraphPad Software, Inc., San Diego, CA, USA). To compare the differences of continuous outcomes, the Wilcoxon rank-sum test was used for two independent-group comparisons; the Wilcoxon signed-rank test was used for paired group comparison and the Kruskal–Wallis test or Friedman test was used for multiple-group comparisons. *p*-values were two-sided, and a *p*-value < 0.05 was considered statistically significant.

## 3. Results

### 3.1. Baseline Participant Characteristics, Risk of COVID-19, Vaccination Status, and Complications during Study

The study had a total of 74 participants enrolled, of which 81% (*n* = 60) were fully vaccinated against SARS-CoV-2 according to CDC guidelines at study entry, and 19% (*n* = 14) were unvaccinated at baseline. Six of the 14 (42%) unvaccinated individuals received some type of SARS-CoV-2 vaccine after study entry. In addition, 29 (39%) of the participants received a third dose (booster) of an mRNA vaccine. The participants’ demographics and other baseline characteristics were collected via a questionnaire applied at each time point by the research staff (Table 1). Of note, 25 of the total 74 participants had self-reported a positive SARS-CoV-2 test result, but none of those 25 reported symptoms that led to serious complications. This included 11 of the 60 vaccinated participants (18%, 95% CI, 0.095–0.304) reporting a positive test during the study. No serious complications from COVID-19 infection or side effects from vaccinations were observed during our study.

### 3.2. SARS-CoV-2 Immune Response 6 Months after Vaccination

**Figure 1:**
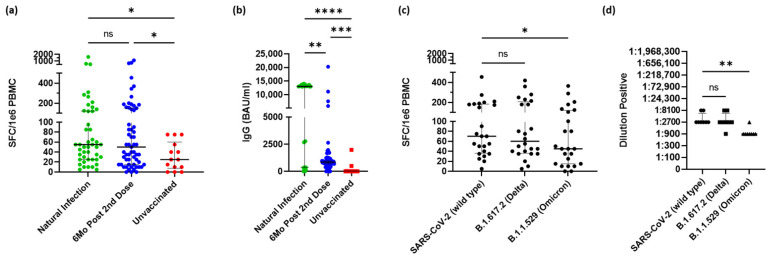
Oncology workers have a comparable immune response to naturally infected individuals within 6 months following a full vaccination regimen but still greater than unvaccinated oncology workers. (**a**) Spike-specific CD4^+^ T-cell response in participants during natural infection (acquired from separate study, *n* = 45), within 6 months of their last dose of a vaccine (*n* = 60) or have never received a vaccine (*n* = 14). (**b**) Spike-specific IgG antibody response from same participants. (**c**) Spike-specific CD4^+^ T-cell response to the wild type, Delta, and Omicron variants within 6 months after second dose of initial vaccination (*n* = 24). (**d**) Spike-specific antibody response to the wild type, Delta, and Omicron variants within 6 months after second dose of initial vaccination. Middle black bar represents median with outer bars representing interquartile range. Kruskal–Wallis test (**a**,**b**) or Friedman test (**c**,**d**) were used for group comparisons. Abbreviations: SFC, spot forming cells; IgG, immunoglobulin G; BAU, binding antibody units; ns, no significance. * *p* < 0.05, ** *p* < 0.01, *** *p* < 0.001, **** *p* < 0.0001.

Vaccinated participants had baseline PBMCs and serum isolated within 6 months following full vaccination (49/60 [82%] were vaccinated 6 months prior, 5/60 [8%] were vaccinated 3–4 months prior, and 6/60 [10%] had unknown vaccination dates). In addition, naturally infected individuals who were unvaccinated from Hillsborough County [13], Florida from a previous study also had PBMCs and serum isolated from whole blood following diagnosis after a positive polymerase chain reaction test, separate from the unvaccinated group. Both groups’ IFN-γ–secreting CD4^+^ T-cells and IgG antibody titers were measured using ELISpot and ELISA, respectively (Figure 1). Confirmation of CD4^+^ T-cells was performed using HLA class I– and class II–blocking antibodies (Supplementary Figure S2). As seen in Figure 1a, no significant difference was detected between the medians of the IFN-γ–secreting T-cells of the naturally infected group (med = 55.0 SFC/10^6^ PBMCs) and the vaccinated group (med = 50.0 SFC/10^6^ PBMCs). However, when compared to the unvaccinated group (med = 25.0 SFC/10^6^ PBMCs), both the naturally infected group and the vaccinated group have statistically significant higher responses (*p* = 0.0168 and 0.0361, respectively). Similarly, as shown in Figure 1b, statistically significant higher IgG antibody titers were detected in both the vaccinated (med = 1187 BAU/mL, *p* = 0.0003) and naturally infected (med = 12,894 BAU/mL, *p* < 0.0001) when compared to the unvaccinated group (med = 0 BAU/mL). Interestingly, a higher antibody response was detected in those naturally infected compared to the vaccinated group (*p* = 0.0083, Figure 1b). In recent variants of concern B.1.617.2 (Delta) and B.1.1.529 (Omicron), the participants who had been vaccinated within 6 months had insignificant differences in CD4^+^ T-cell responses between Delta and the initial SARS-CoV-2 wild-type strain (Figure 1c). Notably, however, the CD4^+^ T-cell response to Omicron (med = 22.50 SFC/10^6^ PBMCs) was significantly lower compared to the wild type (*p* = 0.0304) and similar to the response to the wild type in unvaccinated individuals (med = 25.00 SFC/10^6^ PBMCs). This trend was also noted in antibody titer sensitivity (Figure 1d). No significant difference was noted in T-cell response between manufacturers Pfizer (BNT162b2) and Moderna (mRNA-1273), but Moderna’s mRNA-1273 vaccine did have significantly higher IgG antibody response 6 months after initial vaccination (Supplementary Figure S3).

### 3.3. Booster Dose of mRNA Vaccine Increases Immune Response

**Figure 2:**
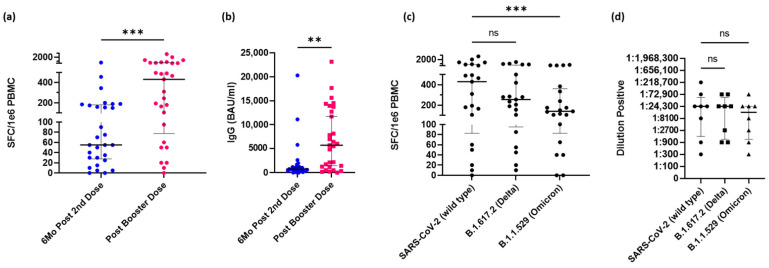
mRNA vaccine Booster dose increases immune response to SARS-CoV-2 spike protein and recent variants of concern in oncology workers. (**a**) Spike-specific CD4^+^ T-cell response in participants within 6 months of their last dose of a vaccine and after they have received a booster dose (*n* = 29). (**b**) Spike-specific IgG antibody response from the same participants. (**c**) Spike-specific CD4^+^ T-cell response to the wild type, Delta, and Omicron variants following a third dose (*n* = 21). (**d**) Spike-specific antibody response to the wild type, Delta, and Omicron variants following a third dose (*n* = 8) Middle black bar represents median with outer bars representing interquartile range. The Wilcoxon signed-rank test (**a**,**b**) or Friedman test (**c**) was used for group comparisons. Abbreviation: SFC, spot forming cells; IgG, immunoglobulin G; BAU, binding antibody units; ns, no significance. ** *p* < 0.01, *** *p* < 0.001.

The vaccinated participants who received a booster dose during the study had both PBMCs and serum isolated within two weeks following their booster dose. We considered any third dose as a booster, irrelevant of dosage amount or manufacturer. Following the booster dose with an mRNA vaccine, IFN-γ–secreting CD4^+^ T-cells increased significantly in response to whole S protein (*p* = 0.0006, Figure 2a). Likewise, IgG antibodies also significantly increased (*p* = 0.0022, Figure 2b). Comparatively, the reactive CD4^+^ T-cells to the Delta variant were not significantly different compared to the wild type but were significantly lower in the Omicron variant compared to the wild type (*p* = 0.0009, Figure 2c). However, antibody titers did not show a significant difference in sensitivity (Figure 2d) Notably, the CD4^+^ T-cell response to Omicron (med = 140 SFC/10^6^ PBMCs) was higher than the response to the wild type observed 6 months after the second dose (med = 55 SFC/10^6^ PBMCs).

### 3.4. Vaccination Increases IFN-γ–Secreting CD4^+^ T-cells and IgG Antibodies

**Figure 3:**
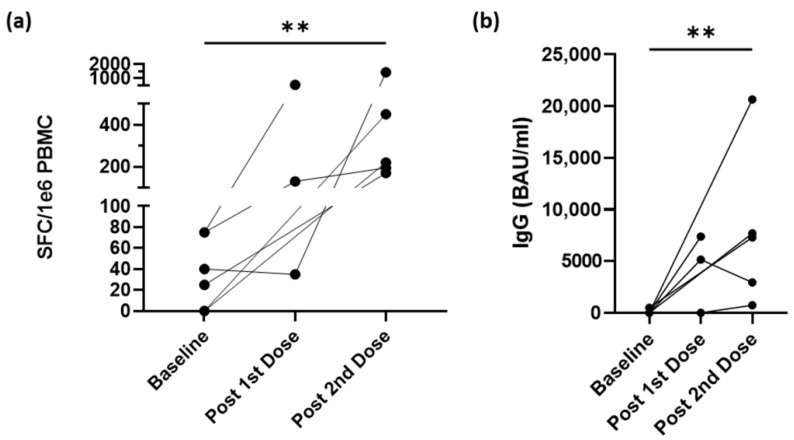
Vaccination increases immune response to SARS-CoV-2 spike protein in oncology workers. (**a**) Spike-specific CD4^+^ T-cell responses measured using IFN-γ ELISpot, from PBMCs isolated before vaccination (baseline), and 2 weeks following additional doses of a mRNA-based vaccine (*n* = 6). (**b**) Spike-specific IgG antibody response measured using ELISA from serum from same participants. Kruskal–Wallis test was used for group comparisons. Abbreviations: IFN-γ, interferon gamma; ELISA, enzyme-linked immunosorbent spot assay; ELISpot, enzyme-linked immunosorbent spot SFC, spot forming cells; IgG, immunoglobulin G; BAU, binding antibody units. **: *p* < 0.01.

Participants who were unvaccinated at study entry and later received at least one dose of a SARS-CoV-2 vaccine had PBMCs and serum isolated from whole blood within two weeks after their doses. Using the PBMCs, we performed an ELISpot assay to measure the response to the whole S protein of SARS-CoV-2 via IFN-γ–secreting CD4^+^ T-cells (Figure 3a). All six participants started below 80 SFC/10^6^ PBMCs and showed at least twofold increases following at least one dose. Using serum collected, IgG titers were measured using ELISA against the whole S protein (Figure 3b). Similar to CD4^+^ T-cell responses, IgG titers markedly increased after at least one dose. Of note, one participant did not have a response after only one dose of the vaccine but did have both a CD4^+^ T-cell and IgG antibody response following the second dose. In general, the mRNA-based vaccines appear to have an increased effect on the CD4^+^ T-cell response consistent with data in Figure 2. Notably, none of these participants had reported testing positive for COVID-19.

### 3.5. Spike-Reactive CD4^+^ T-cells Wane following Booster but Not below Pre-Boosted Levels

**Figure 4:**
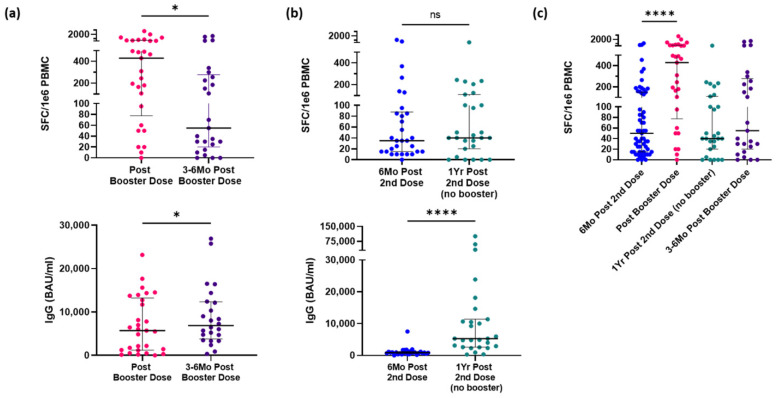
Strong effects of booster wane over time, but not below pre-boosted levels. (**a**) Spike-specific CD4^+^ T-cell (above) and IgG antibody (below) within 2 weeks after participants received a third dose and 3 to 6 months follow-up (*n* = 29). (**b**) Spike-specific CD4^+^ T-cell (above) and IgG antibody (below) within 6 months after participants received last dose of vaccine 1-year follow-up (*n* = 31). (**c**) A summary of spike-specific CD4^+^ T-cell response from participants’ baseline within 6 months of final dose of initial vaccine regimen, response following a third dose (if applicable), and follow-up response 6 months after baseline (no booster) or 3 to 6 months after a third dose. Wilcoxon signed-rank test (**a**,**b**) or Kruskal–Wallis test (**c**) were used for group comparisons. Middle black bar represents median with outer bars representing interquartile range. Comparisons not significant unless otherwise shown. Abbreviations: SFC, spot forming cells; IgG, immunoglobulin G; BAU, binding antibody units; NS, no significance. * *p* < 0.05, **** *p* < 0.0001.

**Figure 5:**
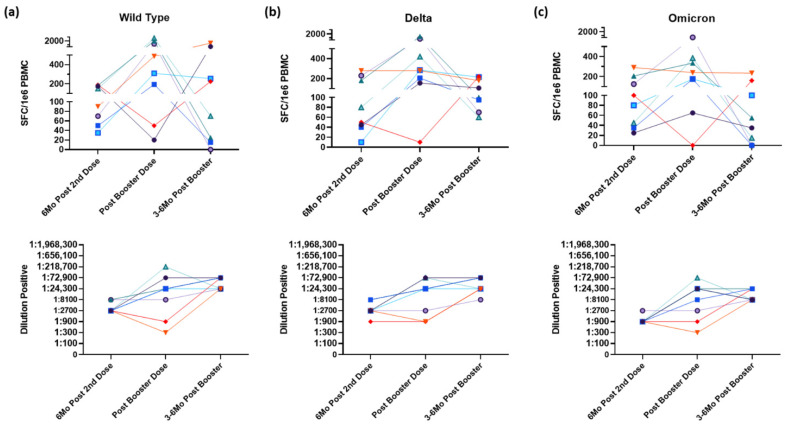
Booster dose raises T-cell and antibody response to both Delta and Omicron variants comparable to unmutated spike protein in individual participants. Spike-specific CD4^+^ T-cell (top) and antibody (below) response to the wild type (**a**), Delta (**b**), and Omicron (**c**) variants 6 months following second dose, 2 weeks after booster and 6 months after booster. Colored lines represent individual participants across all graphs. Abbreviations: SFC, spot forming cells.

In a follow-up collection 1 year after the initial vaccination (3–6 months after booster), all participants, both who received a booster dose or did not, had PBMCs and serum isolated from whole blood. A significant decrease in IFN-γ–secreting CD4^+^ T-cells was seen within 3 to 6 months after their booster dose (*p* = 0.0354, Figure 4a). However, no significant difference was seen in CD4^+^ T-cells response in the group that did not receive a third dose 1 year after their initial vaccination (Figure 4b). Interestingly, when antibodies were measured in these follow-up collections, there was a significant increase in both the group that received a booster and the group that never received a booster (*p* = 0.0491 and *p* < 0.0001, respectively). As summarized in Figure 4c, there is a significant increase (*p* < 0.0001) in CD4^+^ T-cell response following a booster dose of an mRNA vaccine compared to response measured 6 months after initial vaccination but returns to baseline levels as quickly as 3 months later. However, there is no significant difference in response 1 year following the initial vaccination. Additionally, there are no significant differences in spike-specific CD4^+^ T-cell responses to the Delta and Omicron variants compared to the wild type (Figure 4c). Similarly, CD4^+^ T-cell responses and antibody titer sensitivity to the variants also follow the trend of increasing after receipt of a booster dose, but CD4^+^ T-cell responses wane within 6 months (Figure 5). The antibody response remained elevated or increased, similar to the data in Figure 4.

## 4. Discussion

The SARS-CoV-2 pandemic has put healthcare workers at higher risk of infection [17]. Our prospective cohort study aims to monitor the immune responses to the virus in this high-risk population following vaccination against SARS-CoV-2. To do so, 74 healthcare workers in the Tampa, Florida, USA area consented and enrolled to study procedures.

In our comparison with individuals who were infected and unvaccinated at the time of a blood draw collected from another study [13], we noted that CD4^+^ T-cell responses were not significantly different compared to those who were vaccinated within 6 months prior. However, those who were unvaccinated had significantly lower CD4^+^ T-cell responses to either cohort. Not surprisingly, those who were naturally infected had a much higher antibody response in either cohort, as their serum was collected shortly after the infection was confirmed. The cohort that had been vaccinated 6 months prior had measurable antibody responses but were significantly lower than those seen in the naturally infected cohort, most likely due to the time from vaccination. Additionally, the unvaccinated healthcare workers had no measurable humoral response, while still showing a baseline background T-cell response. 

Importantly, our data show the reactivity of CD4^+^ T-cells in response to recent variants of concern: B.1.617.2 (Delta) and B.1.1.529 (Omicron). Omicron is known to have more mutations in the S protein compared to the wild type, which could explain the reduced CD4^+^ T-cell reactivity in participants within 6 months of their initial vaccine regimen [18]. The response in Omicron is more comparable to the T-cell response to the wild type of SARS-CoV-2 in unvaccinated individuals. Spike-specific CD4^+^ T-cells and IgG antibody titers increased markedly following vaccination, whether it was in participants who were receiving their first regimen or those who received third doses (booster). In addition, following a third dose, CD4^+^ T-cell response is higher than the cohort who were naturally infected, while IgG antibody response remained lower than those seen in natural infections, despite increasing otherwise. This increase in CD4^+^ T-cells carried over to the Delta and Omicron variants as well. While the response to Omicron was significantly lower than the wild type following a third dose, it still increased compared to the baseline response following the initial vaccine regimen and is comparable to the response seen in the wild type before boosting.

Our prospective long-term study aims to show the effects of time on both the CD4^+^ T-cell and IgG antibody response after initial vaccination and subsequent booster doses. While both CD4^+^ T-cell and antibody responses increase significantly following a booster dose, this CD4^+^ T-cell response seemingly falls after 3 to 6 months and is comparable to the response seen 6 months following the initial vaccine regimen. Interestingly, in this follow-up timepoint, IgG responses did not decrease and were significantly higher in both the cohort that had received a booster 3 to 6 months prior and the cohort that never received an additional dose and is 1 year out from their last vaccine. Additionally, only three participants in the cohort that had not received boosters had reported positive COVID-19 infections within the study. However, this increase could be due to unreported breakthrough infections caused by the rise in the Omicron variant, especially as it surged in Florida at the time of collections (January–March 2022).

Our data also support the growing body of evidence that vaccination increases the spike-specific antibody response to SARS-CoV-2 [8,9,10]. However, the longevity of the response of CD4^+^ T-cells and if a higher T-cell response confers protection from COVID-19 is still being investigated. We assessed whether the CD4^+^ T-cell response is longer lasting than the antibody response. In those participants who were originally unvaccinated at enrollment, they did not have a measurable IgG antibody response until after they received a vaccine. Likewise, these participants’ spike-specific CD4^+^ T-cell responses also increased, despite some having a pre-existing response, most likely due to cross-reactivity with other seasonal coronaviruses [19].

We acknowledge the limitations of having a small unvaccinated cohort and relying on the self-reporting of positive tests and symptoms. While CD4^+^ T-cell responses may be sufficient before additional doses of vaccine, boosting may be necessary to confer protection against the Omicron variant and other future variants of concern. Notwithstanding the clear evidence that mRNA vaccines are associated with both humoral and cellular responses, 11 out of the 60 vaccinated participants in our study were diagnosed with COVID-19 infection after study entry (AR 18%, 95% CI, 0.095–0.304). Of note, most participants in our study had patient-facing job posts (Table 1). Collectively, these findings suggest that healthcare workers should adhere to contact precautions and receive vaccinations; in parallel institutions, adequate screening of patients for possible infection should be performed to reduce the risk of COVID-19 infections. In addition, future variants of concern can be studied using this pipeline to compare responses in the population to the wild type to determine immunogenicity and possible protection, in addition to justifying the need for additional or modified booster doses. We acknowledge the limitations in choosing to use MHC blocking within the ELISpot platform as the determinant for CD4^+^ cellular responses. However, where similar tests utilizing flow cytometry and identifying more specific cell populations (e.g., CD4^+^ versus CD8^+^ responses, memory populations, etc.) would be more appropriate, the present study was limited by the amount of cells collected from the study participants, limiting us to using the platforms chosen. Future considerations for this limitation will be made for follow-up time points. Despite the shortcomings, we had no evidence of severe cases of COVID-19 in any of those who were vaccinated, further adding to the evidence that while some participants reported testing positive, vaccination can prevent severe outcomes from infection [8,9,10]. Follow-up on the length of the response as well as response to new and existing variants of concern will be measured in subsequent time points for the remainder of this prospective study, which will further elucidate the longevity and effectiveness of the initial vaccination and subsequent doses. Likewise, neutralizing antibodies can also be investigated within these cohorts following subsequent booster doses, which may help better understand the importance of the antibodies measured in this study. Determinations of Spike-specific CD4^+^ T-cell responses may be useful to determine the need for additional booster doses for future waves of variants of concern.

## 5. Conclusions

In our prospective cohort study, we present preliminary evidence that vaccination against SARS-CoV-2 leads to both IgG and CD4^+^ T-cell responses across different variants. These responses wane over time suggesting the importance of serial vaccination for high-risk populations.

## Figures and Tables

**Table 1:** Summary of participants in the study. Data are N (%) unless otherwise noted.

	Vaccinated Participant N = 60 (81%)	Unvaccinated Participant N = 14 (19%)	Total N = 74 (100%)
Age (median and range)	42.5 (24–70)	37 (25–55)	42 (24–70)
Gender			
Male	12 (20%)	0 (0%)	12 (16%)
Female	48 (80%)	14 (100%)	62 (83%)
Race			
White	52 (87%)	12 (86%)	64 (86%)
Black or African American	1 (2%)	2 (14%)	3 (4%)
Asian	5 (8%)	0	5 (7%)
Some other race	2 (3%)	0	2 (3%)
Ethnicity			
NonHispanic or Latino	50 (83%)	10 (71%)	60 (81%)
Hispanic or Latino	6 (10%)	4 (29%)	10 (14%)
Other	1 (2%)	0	1 (1%)
Unknown	3 (5%)	0	3 (4%)
Occupation			
Patient-facing	52 (87%)	13 (93%)	65 (88%)
Nonpatient-facing	8 (13%)	1 (7%)	9 (12%)
Vaccine manufacturer			
Moderna	36 (60%)	4 (29%)	40 (54%)
Pfizer	22 (37%)	1 (7%)	23 (31%)
Other	2 (3%)	NA	2 (3%)
Third dose/booster	29 (48%)	NA	29 (39%)
Reported positive COVID-19 test *	19 (32%)	6 (43%)	25 (34%)
Before enrollment	8 (13%)	5 (36%)	13 (18%)
During study	11 (18%)	1 (7%)	12 (12%)

Abbreviation: NA, not applicable. * As determined by self-reported results from antibody or polymerase chain reaction test at any point after enrollment.

## Data Availability

The data that support the findings of this study have been originated by Flatiron Health, Inc. These de-identified data may be available upon request.

## Supplementary Materials

The following supporting information can be downloaded at: https://www.mdpi.com/article/10.3390/vaccines10111931/s1. Supplementary Figure S1. Schematic diagram of cohorts. Cohorts are separated by vaccinated status along with timing of collections. Supplementary Figure S2. T-cell response to whole spike protein is mediated by MHC class II. Supplementary Figure S3. Comparison of T-cell and IgG response between Moderna mRNA-1273 and Pfizer-BioNTech BNT162b2 6 months after initial vaccination.
