# Supplementary material for: Long-Term CD4+ T-Cell and Immunoglobulin G Immune Responses in Oncology Workers following COVID-19 Vaccination: An Interim Analysis of a Prospective Cohort Study
Source: Vaccines (Basel). 2022 Nov 15;10(11):1931. doi: 10.3390/vaccines10111931 (PMC9696551; doi:10.3390/vaccines10111931)
Supplement: Supplementary file 1 [file vaccines-10-01931-s001.zip › vaccines-2012387-supplementary.pdf]

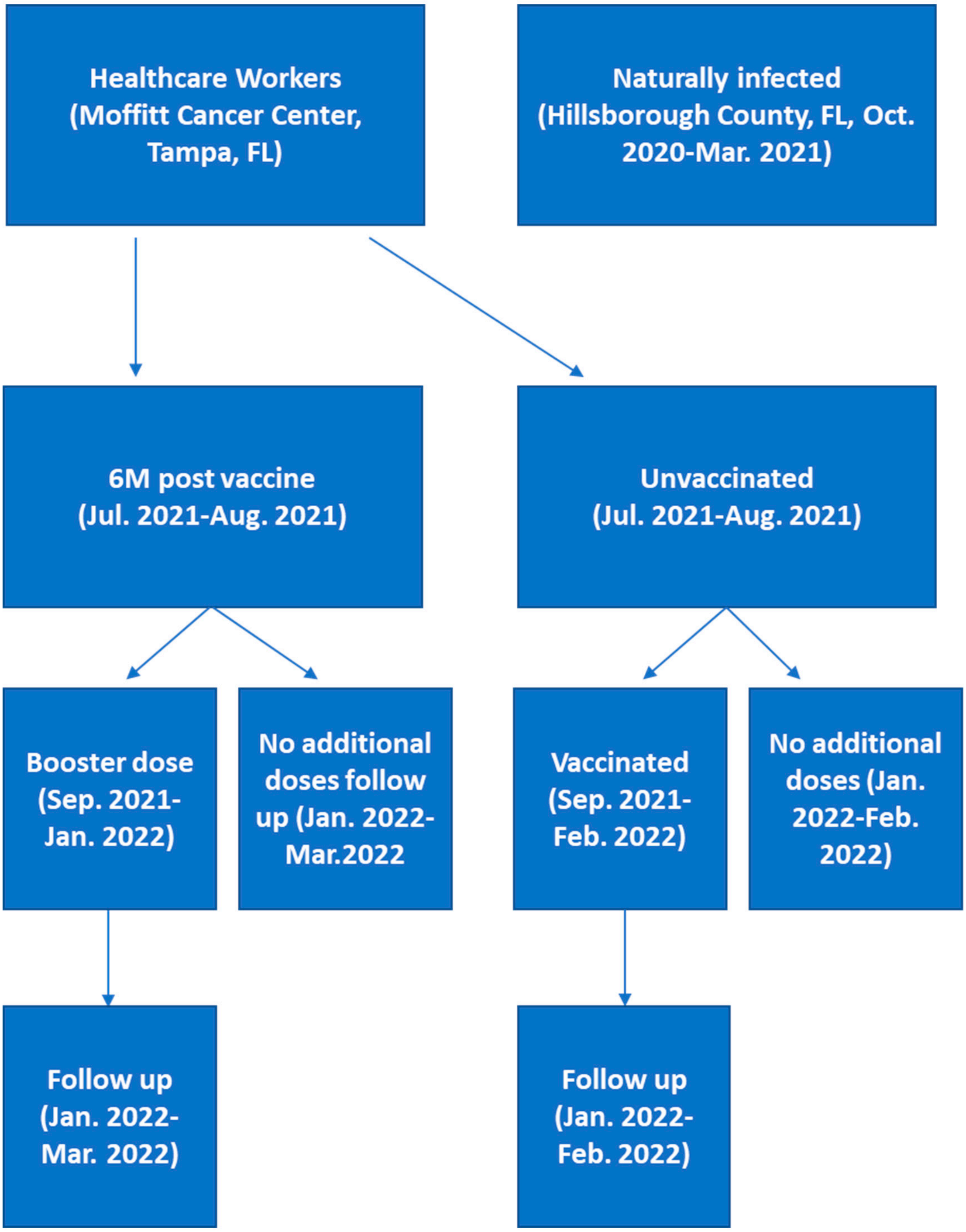

Supplementary Figure S1. Schematic diagram of cohorts. Cohorts are separated by vaccinated status along with timing of collections.

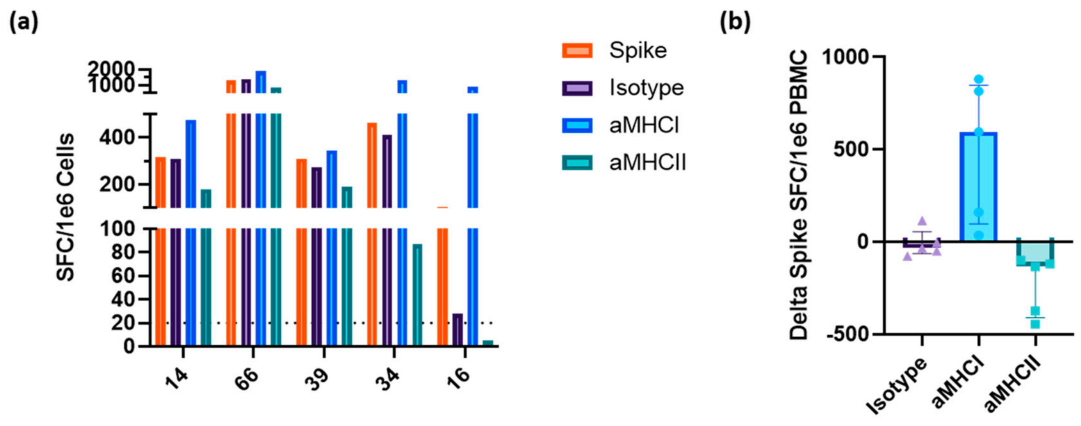

Supplementary Figure S2. T-cell response to whole spike protein is mediated by MHC class II.

a) Response to spike protein before and after incubation with either MHC class I– or MHC class II–blocking antibodies or isotype control. b) Difference in response when in the presence of Isotype, MHC class I–blocking, or MHC class II–blocking compared to response without expressed as Delta SFC.

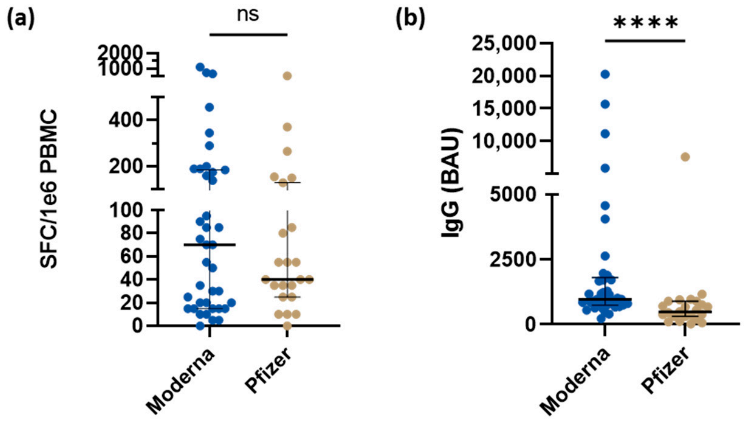

Supplementary Figure S3. Comparison of T-cell and IgG response between Moderna mRNA-1273 and Pfizer-BioNTech BNT162b2 6 months after initial vaccination.

a) Spike-specific CD4+ T-cell response in participants who received either mRNA-123 (n = 39) or BNT162b2 (n = 23) 6 months following initial dosing regimen. b) Spike-specific IgG antibody response from the same participants. Wilcoxon signed-rank test was used for group comparisons. Middle black bar represents median with outer bars representing interquartile range. Abbreviations: IgG, immunoglobulin G; ns, no significance. **** *p < .0001*
